# Supplementary material for: Combination of the Glutaminyl Cyclase Inhibitor PQ912 (Varoglutamstat) and the Murine Monoclonal Antibody PBD-C06 (m6) Shows Additive Effects on Brain Aβ Pathology in Transgenic Mice
Source: Int J Mol Sci. 2021 Oct 30;22(21):11791. doi: 10.3390/ijms222111791 (PMC8584206; doi:10.3390/ijms222111791)
Supplement: Supplementary file 1 [file ijms-22-11791-s001.zip › ijms-1390312-supplementary.pdf]

International Journal of
Molecular Sciences

MDPI

Supplementary material

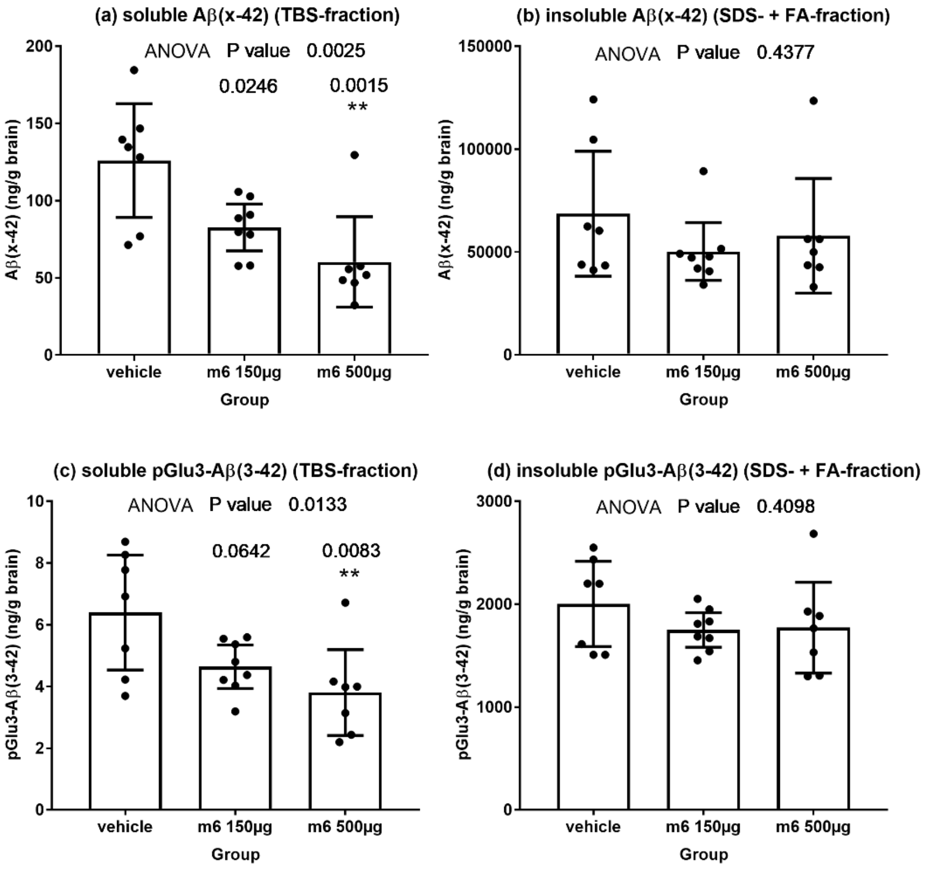

Figure S1: Comparison of brain Aβ levels of 12 months old hAPPsl×hQC mice after 16-week treatment with different m6 antibody doses (ELISA).

Aβ(x–42) (a, b) and pGlu3-Aβ(3–42) (c, d) concentration in soluble (a, c) and insoluble (b, d) brain fractions of 12 months old $hAPP_{sl}$×hQC mice after 16 weeks of treatment were determined by specific ELISAs. Dots represent individual levels. Bars and whiskers represent mean ± 95% CI. ANOVA p-values are given on top of the graphs. Numbers above the bars represent adjusted p-values of Dunnett's post-hoc comparison with the vehicle control. The analysis revealed a dose dependent significant decrease of total Aβ42 and pGlu3-Aβ42 by the m6 treatment in the TBS fractions.

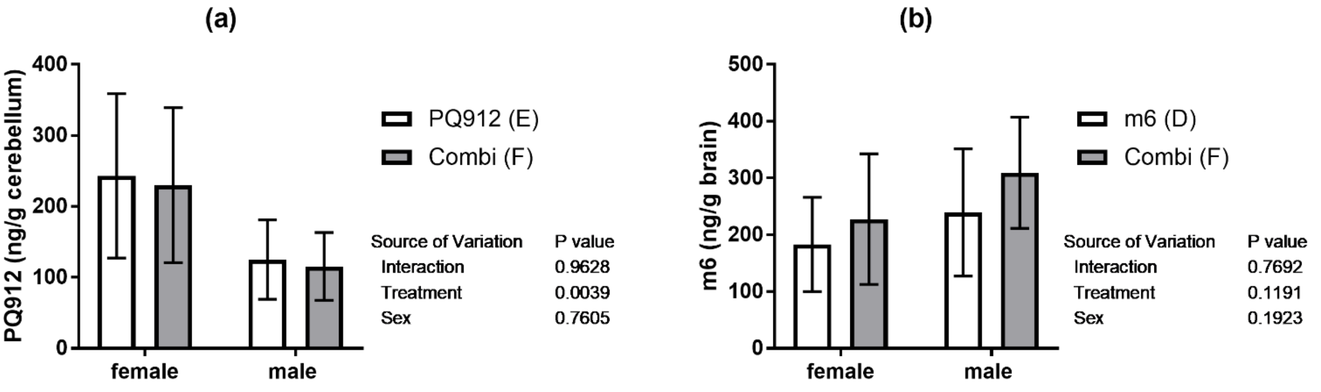

Figure S2: Brain levels of PQ912 and m6 antibody (combination experiment)
Brain levels (mean ± 95% CI) of PQ912 (a) and m6 antibody (b) in single (white) and combination (gray) treatment groups in male and female hAPPsl×hQC mice

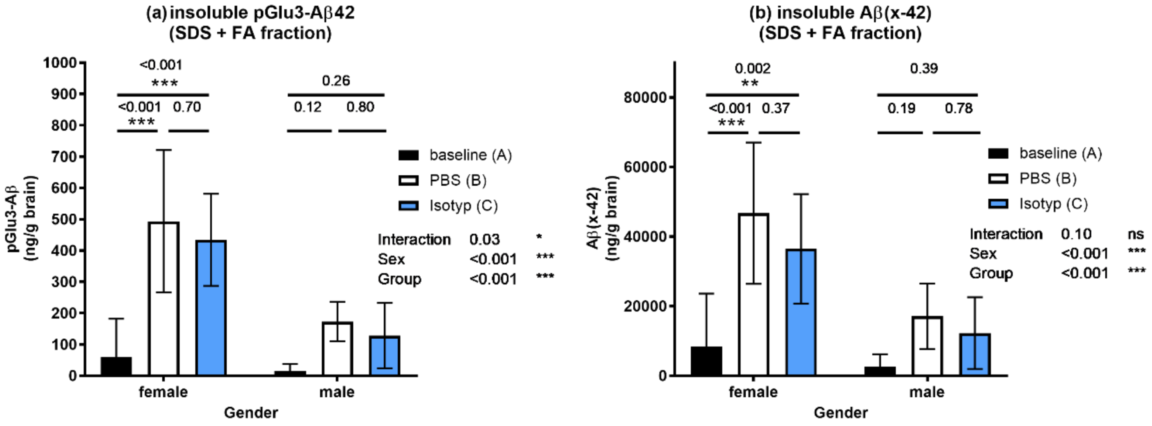

Figure S3: Development of Aβ pathology in female and male hAPPsl×hQC mice during the 16-week treatment period. Aβ levels (a: pGlu3-Aβ42, b: Aβ42) in insoluble brain fraction were determined byELISA in female and male hAPPslxhQC mice from baseline (9 month, black) over 16 weeks of treatment with vehicle (PBS) control (white) or Isotype antibody control (blue). Two-way-ANOVA indicates a strong effect of gender and treatment on brain Aβ levels (means ± 95% CI). A significant difference to baseline was only seen in females. Increase of Aβ levels in males were much lower and did not reach significance.

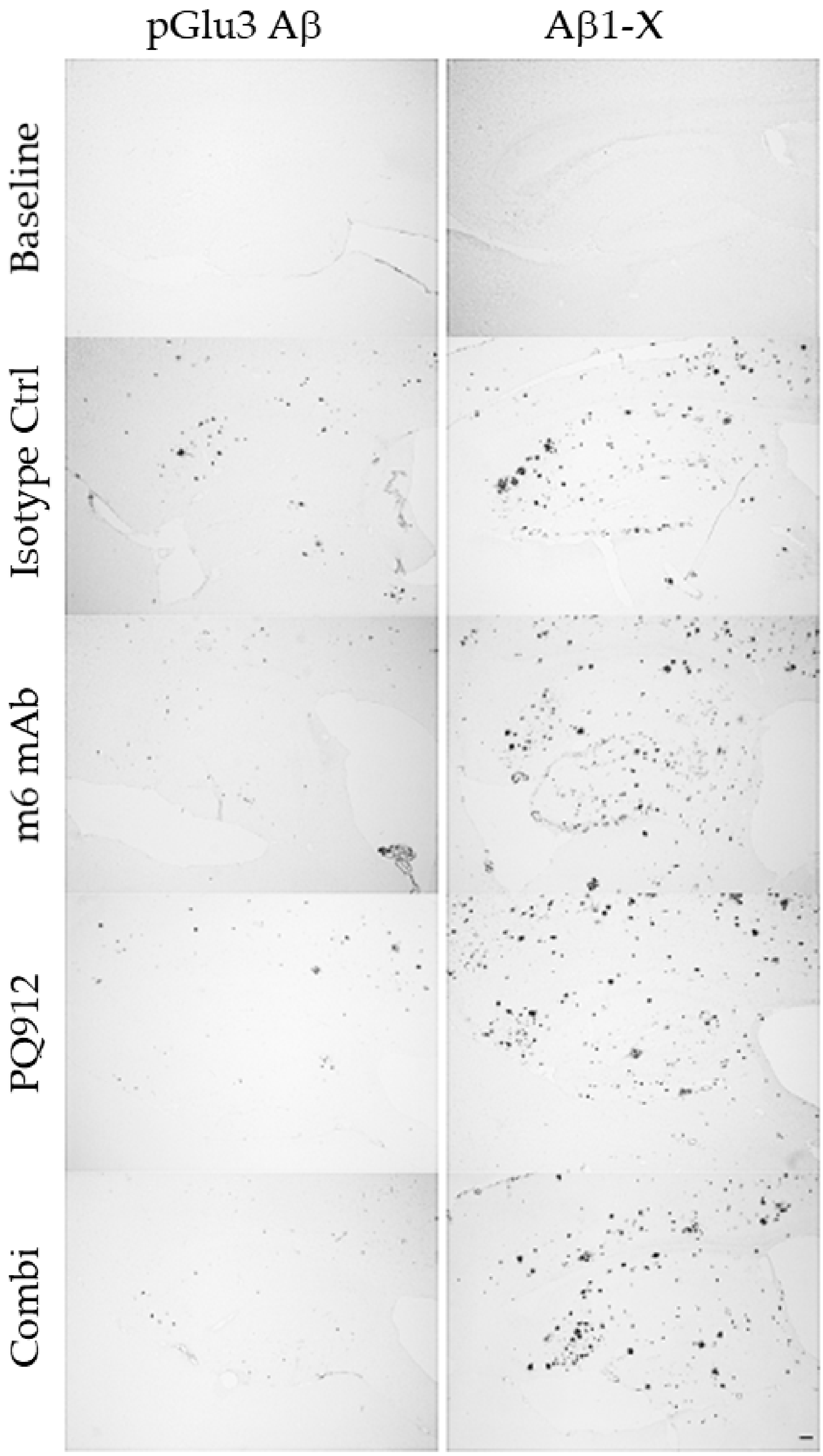

Figure S4: Representative images of brain slices stained for pGlu3-Aβ with K17 antibody(left panel) and Aβ(1–x) with 82E1 antibody (right panel). Scale bar at lower left represents 100 µm and is representative for all subfigures.
